# Pentylenetetrazole-induced seizures in developing rats prenatally exposed to valproic acid

**DOI:** 10.7717/peerj.2709

**Published:** 2016-11-29

**Authors:** Angel A. Puig-Lagunes, Jorge Manzo, Luis Beltrán-Parrazal, Consuelo Morgado-Valle, Rebeca Toledo-Cárdenas, Maria-Leonor López-Meraz

**Affiliations:** 1 Doctorado en Investigaciones Cerebrales, Universidad Veracruzana, Xalapa, Veracruz, Mexico; 2 Centro de Investigaciones Cerebrales, Universidad Veracruzana, Xalapa, Veracruz, Mexico

**Keywords:** Epilepsy, Convulsions, Autism, Valproic acid, Pentylenetetrazole

## Abstract

**Background:**

Epidemiological evidence indicates epilepsy is more common in patients with autism spectrum disorders (ASD) (20–25%) than in the general population. The aim of this project was to analyze seizure susceptibility in developing rats prenatally exposed to valproic acid (VPA) as autism model.

**Methods:**

Pregnant females were injected with VPA during the twelfth embryonic day. Seizures were induced in fourteen-days-old rat pups using two models of convulsions: pentylenetetrazole (PTZ) and lithium-pilocarpine (Li-Pilo).

**Results:**

Two subgroups with different PTZ-induced seizure susceptibility in rats exposed to VPA were found: a high susceptibility (VPA+) (28/42, seizure severity 5) and a low susceptibility (VPA−) (14/42, seizure severity 2). The VPA+ subgroup exhibited an increased duration of the generalized tonic-clonic seizure (GTCS; 45 ± 2.7 min), a higher number of rats showed several GTCS (14/28) and developed status epilepticus (SE) after PTZ injection (19/27) compared with control animals (36.6 ± 1.9 min; 10/39; 15/39, respectively). No differences in seizure severity, latency or duration of SE induced by Li-Pilo were detected between VPA and control animals.

**Discussion:**

Prenatal VPA modifies the susceptibility to PTZ-induced seizures in developing rats, which may be linked to an alteration in the GABAergic transmission. These findings contribute to a better understanding of the comorbidity between autism and epilepsy.

## Introduction

Two neurological conditions that importantly affect the pediatric population are epilepsy and autism spectrum disorder (ASD) or autism. Epilepsy is defined as a disorder of the brain characterized by an enduring predisposition to generate epileptic seizures (Fisher et al., 2014). ASD is a pervasive developmental disorder characterized by three main behavioral symptoms including social deficits, impaired communication, and stereotyped, repetitive behaviors (Rapin & Tuchman, 2008). Epidemiological studies refer the existence of comorbidity between autism and epilepsy. In fact, different reports show that up to a third of patients with autism have epilepsy (Bolton et al., 2011; Danielsson et al., 2005; Gabis, Pomeroy & Andriola, 2005; Hara, 2007; Hughes & Melyn, 2005). Some reports suggest that epilepsy is the most common central nervous system disorder associated with autism; autistic patients with severe mental retardation, cognitive impairment and tuberous sclerosis have a higher risk of developing epilepsy (Bolton et al., 2011; Danielsson et al., 2005; Gabis, Pomeroy & Andriola, 2005). The incidence of epilepsy among the autistic population seems to follow a bimodal distribution with a first peak at age between 1–5 years old, then a second peak between prepuberty and adolescence (children more than 10 years old) (Bolton et al., 2011; Danielsson et al., 2005; Gabis, Pomeroy & Andriola, 2005; Hara, 2007). Generalized tonic-clonic seizure (GTCS) is one of the most common type of seizures observed in the ASD population (Hara, 2007; Hughes & Melyn, 2005; Rapin & Tuchman, 2008).

Valproic acid (VPA) is a drug used to treat epilepsy, migraine and bipolar disorder. Clinical studies in the last 40 years have shown that exposure to VPA during specific pregnancy periods is associated with an increment of approximately three times the rate of major anomalies in the neonates, including skeletal malformation and behavioral impairment (Christianson, Chesler & Kromberg, 1994; Fried et al., 2004; Meador et al., 2008). Also, it has been demonstrated that VPA monotherapy during the first trimester of pregnancy resulted in greater incidence of ASD or key symptoms of ASD (Bromley et al., 2008; Bromley et al., 2013; Christianson, Chesler & Kromberg, 1994; Moore et al., 2000; Williams & Hesh, 1997). VPA affects the closing of neural tube during the neurodevelopmental process by inhibiting the histone deacetylase (Arndt, Stodgell & Rodier, 2005; Phiel et al., 2001).

Rodier et al. (1996) developed an animal model of autism induced by prenatal exposure of rats to VPA. This model has been validated in terms of the similarities to human autistic symptoms, such as altered sensitivity to stimuli, lower exploratory activity and decreased social behaviors (Kim et al., 2011; Kim et al., 2013; Rodier et al., 1996; Schneider & Przewlocki, 2005). The goal of this study was to analyze the susceptibility of fourteen-days-old rat pups exposed prenatally to VPA (experimental model of autism) to two experimental models of acute seizures: pentylenetetrazole (PTZ) convulsions or lithium-pilocarpine (Li-Pilo) *status epilepticus* (SE) (Turski et al., 1989; Velisek et al., 1992). These models were chosen since GTCS and SE have been observed in patients with autism or during childhood in the general population (Bolton et al., 2011; DeLorenzo et al., 1996; Govoni et al., 2008; Hara, 2007; Hughes & Melyn, 2005). We used fourteen-days-old rat pups since the brain maturity at this age (12–18 days-old rats) has been suggested to be the equivalent to the infant/toddler human brain (Haut, Velísková & Moshé, 2004). We hypothesized that prenatal VPA will increase seizure susceptibility in infant rats.

## Materials and Methods

### Animals

Adult male and female Wistar rats from our local breeding colony (Centro de Investigaciones Cerebrales, Universidad Veracruzana), were maintained in a vivarium on a 12:12 h circadian cycle (lights on at 08:00), at 23–25 °C temperature and 60–70% relative humidity, with free access to water and food (Rismart). All experiments were conducted during the light period. Conducting daily vaginal smears corroborated the fertility of females; when females were in proestrus, they were placed overnight with a sexually experienced male. The following morning, vaginal smears were collected and analyzed; if spermatozoa were found in the morning, this was reported as the first day of pregnancy. Pregnant females were housed individually during the study. Experiments were approved by a Committee of Graduate Program in Brain Research, Centro de Investigaciones Cerebrales, Universidad Veracruzana. Animal treatment and maintenance were conducted in accordance with the official Mexican specifications for the production, care, and use of laboratory animals (NOM-062-ZOO-1999) as well as the National Institutes of Health guidelines on the care and use of laboratory animals (National Research Council of the National Academies, 2011).

### Administration of valproic acid to pregnant rats

Valproic acid was purchased as the sodium salt (sodium valproate, Sigma, St. Louis, MO, USA) and dissolved in 0.9% saline for a concentration of 250 mg/mL. Females received a single intraperitoneal injection of 600 mg/kg on embryological day 12.5 (Schneider & Przewlocki, 2005); control rats were treated with physiological saline during the same embryonic day (SS group). Females were housed individually and were allowed to raise their own litters.

### Generalized tonic-clonic seizures induced by pentylenetetrazole

To determinate the susceptibility to seizures induced by PTZ, convulsions were induced in 81 rats on the fourteenth postnatal day (P14). The control group was composed of 22 male and 17 female rat pups for a total of 39 rats; the VPA group was composed of 29 male and 13 female rat pups for a total of 42 rats. PTZ (Sigma, St. Louis, MO, USA) was dissolved in 0.9% saline and administered intraperitoneally at a dose of 80 mg/kg. After PTZ injection, rats were placed in a plexiglass acrylic cage and seizure behavior was recorded until convulsions stopped and rats had totally recovered. The severity of seizures (intensity of seizures) was measured according to scale proposed by Pohl & Mares (1987): 0- no changes in behavior; 1- isolated myoclonic jerks; 2- atypical minimal seizures (unilateral, incomplete); 3- full clonic seizure; 4- pattern of tonic-clonic seizures with a suppression of tonic phase; 5- full GTCS.

### Status epilepticus induced by lithium-pilocarpine

*Status epilepticus* was defined as near continuous seizure activity lasting over 30 min (Wasterlain & Chen, 2006). SE was induced with Li-Pilo in P14 rat pups from both genders. Seizures were produced in 29 control rats (11 males and 18 females) and in 22 rats exposed to VPA (17 males and five females). Rats were injected intraperitoneally with lithium chloride (3 mEq/kg; Sigma, St. Louis, MO, USA) 20 h before convulsions were induced by the subcutaneous injection of 100 mg/kg of pilocarpine hydrochloride (Sigma, St. Louis, MO, USA). Then, rats were placed in a plexiglass acrylic cage for recording seizure behavior. Seizure severity (intensity of seizures) was measured according a slight modification of Haas, Sperber & Moshe (1990) scale as proposed by other authors (Suchomelova et al., 2006; Suchomelova et al., 2015): 0- Behavioral arrest; 1- Mouth clonus; 2- Head bobbing; 3- Unilateral forelimb clonus; 4-Bilateral forelimb clonus; 5- Bilateral forelimb clonus and falling over with or without rearing; 6- Wild running and jumping with vocalizations.

### Behavioral parameters analyzed

For both experimental models of seizures, seizure susceptibility was analyzed considering the following parameters: seizure occurrence, seizure severity (according to the behavioral scale), latency and duration of behavioral seizures, number of animals displaying SE and death due to seizures. All experiments were video-recorded in order to identify more precisely the parameters indicated above.

### Statistical analysis

Normality and homoscedasticity of data were evaluated by using respectively the D’Agostino & Pearson omnibus normality and the Bartlett’s test, respectively. Data displaying equal variances were analyzed by a Student’s *t*-test and reported as the mean ± standard error of the mean (S.E.M.); data that do not meet the criteria of normality were analyzed by a Mann Whitney *U*-test and expressed as the median ± interquartile range. To determine the difference between two proportions, it was used a proportion test. Two-sided statistical testing was performed in all analyses. Statistical significance for all comparisons was considered when *p* < 0.05. Statistical analyses were performed using the Statistica (version 7) or Graphpad Prism (version 6.0d) software.

## Results

### Effect of VPA exposure on birth rate and body weight

Prenatal exposure to VPA decreased up to 30% the rate of birth (*p* < 0.005) and the number of pups per litter (6.3 ± 0.9) when compared with control rats (9 ± 0.7) (five VPA litters, six Saline litters). The proportion of female and male pups per litter was different in both experimental groups; in the VPA group the proportion of females was lower (18/64) than those of males (46/64, *p* < 0.001). Litters from the control group did not show differences in the proportion of rats per gender (51% females 35/68 vs 49% males 33/68, *p* = 0.86).

When the body weight of VPA rats from both genders was measured on 14 postnatal day, the VPA group showed lower body weight (*n* = 42, 24.5 ± 8.8 g) than the control group (n = 39, 29.6 ± 4.9 g) (Mann–Whitney *U* = 248, *p* < 0.0001). This effect was observed both in males (23 ± 8.1 g; Mann–Whitney *U* = 184, *p* < 0.0001) and females (22.7 ± 0.8 g; *t* = 5.15, *p* < 0.0001) rats exposed to VPA in comparison with those from the control group (respectively 28.7 ± 4.7 and 27.3 ± 0.47 g).

Prenatal exposure to VPA produced congenital abnormalities in the pups, which were evident in 61% of the rats. The most common abnormality was a crocked tail with different grades of severity. Similar abnormalities were not detected in rats from the control group.

### Seizures induced by PTZ

**Figure 1:**
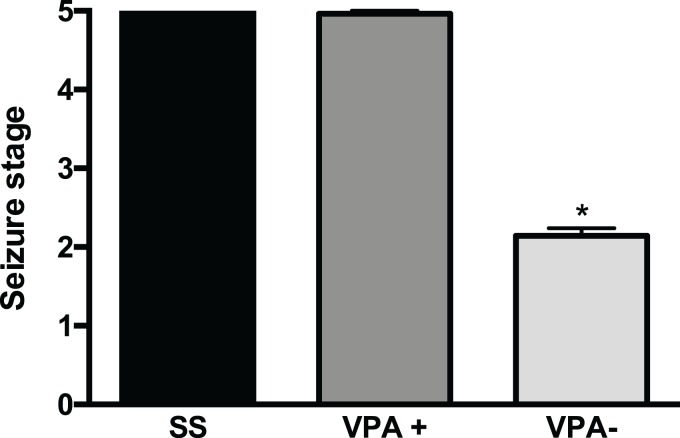
Severity of convulsions induced by pentylenetetrazole (PTZ) in rats prenatally exposed to valproic acid (VPA). Two subgroups with different PTZ-induced seizure severity were observed: the high seizure susceptibility subgroup (VPA+) and the low seizure susceptibility subgroup (VPA−). Data are represented as the mean ± S.E.M. and were analyzed with a Student’s *t*-test. *p < 0.001 vs SS group and VPA+ subgroup.

After a careful analysis of the convulsive behavior of rats prenatally treated with VPA, we found two subgroups with different PTZ-induced seizure severity (*p* < 0.001) (Fig. 1). The first subgroup was termed VPA+ (28/42, corresponding to 67% of VPA rats) and had seizures scored as 4.9 ± 0.04 and corresponding to GTCS (Ninety six percent of VPA+ rats (27/28) had seizure severity scored as 5). Seizure severity in the VPA+ subgroup was similar to that from the control group 5 ± 0.0 (*t* = 1.18, *p* = 0.24). The VPA+ subgroup was mainly made of male rats (20 males and eight females). The second subgroup was termed VPA− (14/42, corresponding to 33% of VPA rats) and had an average seizure severity of 2.1 ± 0.1, corresponding to myoclonus and minimal seizures. Seizure severity was smaller in the VPA− subgroup than in the control and the VPA+ groups (*p* < 0.001) (Fig. 1; Table 1). It is important to mention that the body weight at postnatal day 14 was similar in the VPA+ (23.8 ± 0.8 g) and VPA− (21.2 ± 1.2 g) rats (*t* = 1.790, *df* = 40; *p* = 0.0809). The latency to myoclonus was similar in the VPA+ (54.05 ± 5.2 s) and control groups (50 ± 3.2 s) (*t* = 0.3488, *p* = 0.7286). However, the latency to myoclonus was higher in the VPA− subgroup (108 ± 71.5) than in the control group (53 ± 22) (*p* < 0.0001; Mann–Whitney *U* = 54). Latency to myoclonus was higher in the VPA− subgroup (108 ± 71.5) than in the VPA+ subgroup (50 ± 36.9) (*p* = 0.0003; Mann–Whitney *U* = 42).

**Table 1:** Number of rats displaying seizures after injection with pentylenetetrazole.

Seizures	SS group (n = 39)	VPA group (n = 42)	
		VPA+ subgroup (n = 28)	VPA− subgroup (n = 14)
Seizure stage 2	0	0	12*
Seizure stage 3	0	0	2
Seizure stage 4	0	1	0
Seizure stage 5	39	27*	0
SE (after seizures stage 5)	15	19*	0

**Notes:**

VPA, valproic acid; VPA+, subpopulation of rats treated with VPA with high seizure susceptibility; VPA−, subpopulation of rats treated with VPA with low seizure susceptibility. Seizure severity was evaluated considering the Pohl & Mares (1987). Data were analyzed with a proportion test.

* *p* < 0.05 vs control group and VPA+ or VPA−.

In the VPA+ subgroup a larger number of rats had several GTCS (14/28; *p* < 0.05) and developed SE (19/28; *p* < 0.05) in comparison with the control group (10/39 and 15/39, respectively). VPA+ rats had increased latency to the first GTCS (1.6 ± 0.9 min, Mann–Whitney *U* = 267.5, *p* < 0.001) in comparison with the control group (1 ± 0.5 min). The number of rats that survived to the GTCS or SE (did not die due to seizures) was higher in the VPA+ subgroup (10/27; *p* < 0.001) than in the control group (6/39), i.e., only 63% (17/27) of VPA+ rats died after seizures whereas 85% (33/39) of control rats died after seizures.

**Figure 2:**
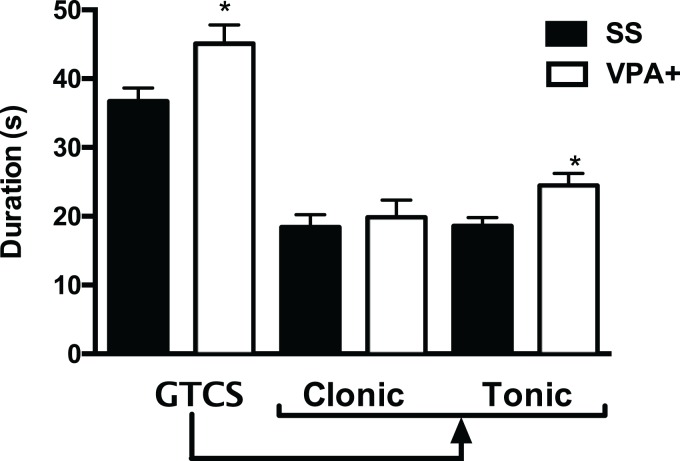
Duration of the generalized tonic-clonic seizure (GTCS) induced by pentylenetetrazole, as well as of its clonic and tonic phases in the subpopulation of rats treated with valproic acid with high seizure susceptibility (VPA+). Data are represented as the mean ± S.E.M. and were analyzed with a Student’s *t*-test. SS, saline group. *p < 0.01 vs SS group.

Results showed that the duration of GTCS was increased in the VPA+ group (45 ± 2.7 s; (*t* = 2.54, *p* < 0.05) when compared with the control group (36.6 ± 1.9 s). When the clonic and tonic phases of the GTCS seizure were separated, data showed that the duration of the tonic phase was increased in VPA+ rats (24.8 ± 1.7 s; *t* = 3.01, *p* = 0.003) compared with control rats (18.5 ± 1.2 s). However, the duration of the clonic phase was similar in VPA+ and control rats (19.8 ± 2.5 and 18.3 ± 1.8 s, respectively; *t* = 0.469, *p* = 0.640) (Fig. 2). Duration of SE was similar in VPA+ and control groups (67.2 ± 5.1 and 66 ± 4.2 min, respectively; *t* = 0.181, *p* = 0.856).

When GTCS duration was evaluated considering the gender, results showed that seizure duration was higher in VPA+ males (47.6 ± 3.3 s; *t* = 3.01, *p* = 0.004) than in control males (34.5 ± 2.7 s). The tonic phase of this seizure lasted more in VPA+ males (26.6 ± 2.6 s; *t* = 3.18, *p* = 0.002) than in control males (18.3 ± 1.6 s), whereas the duration of the clonic seizure was similar in both groups (20.5 ± 2.9 s for VPA+ males and 16.2 ± 2.4 s for control males; *t* = 1.12, *p* = 0.267). Duration of SE was similar in VPA+ males (62 ± 6.1 min; *t* = 0.43, *p* = 0.670) when compared with control males (66 ± 7.3 min). Regarding the females rats, results showed no differences between VPA+ and control rats in the duration of the GTCS (38.8 ± 3.8 and 39.1 ± 2.5 s, respectively; *t* = 0.118, *p* = 0.907) or clonic (18.1 ± 5.2 and 21.1 ± 2.7 s, respectively; *t* = 0.561, *p* = 0.580) and tonic (20.7 ± 3.4 and 18.8 ± 1.8 s, respectively; *t* = 0.561, *p* = 0.580) phases of the GTCS.

### Status epilepticus induced by lithium-pilocarpine

All rats from both experimental groups developed SE. Seizure severity in the VPA group (6 ± 0; *t* = 1.49, *p* = 0.141) was similar to the control group (5.8 ± 0.10). Latency to SE (13 ± 1 min, *t* = 0.445, *p* = 0.660) and duration of SE was similar in the VPA (242.8 ± 16 min; *t* = 1.087, *p* = 0.282) and control groups (12.1 ± 1.2 and 268 ± 17 min, respectively). VPA as well as control rats tolerated well the pilocarpine-induced SE without mortality. No additional differences in SE behavior were observed between experimental groups related to gender.

## Discussion

In the present study, we showed that prenatal exposure to VPA produces two P14 rat subpopulations with different PTZ-induced seizure susceptibility. The VPA+ subgroup showed increased duration of GTCS, particularly of the tonic phase, a higher number of GTCS and progression of acute seizures to SE; whereas the VPA- subgroup only displayed myoclonic seizures. Lithium-pilocarpine-induced SE in VPA rats was similar to the control group.

In this study, we observed that the main congenital abnormality following prenatal exposure to VPA is the presence of a crocked tail in the pups. This observation is similar to previous reports from others (Favre et al., 2013; Foley et al., 2012; Kim et al., 2011; Kim et al., 2013) and our laboratory (Saft et al., 2014; Puig-Lagunes et al., 2015), which confirms that our model is reproducible.

Our results demonstrate changes in the susceptibility to seizures induced by PTZ in rat pups exposed prenatally to VPA, an experimental model of autism. A previous study reported that 4-week-old rat exposed to VPA at E12 displayed lower electroshock seizure thresholds than control rats (Kim et al., 2011). These authors suggest that exposure to VPA at this embryonic stage produces a reduced inhibitory function of the brain similar to autistic patients (Kim et al., 2011). A recent study also demonstrated that *Xenopus laevis* tadpoles exposed to VPA have increased PTZ-induced seizure susceptibility, which was associated with hyperconnected neural networks in the optic tectum, an increased excitatory and inhibitory synaptic drive and elevated levels of spontaneous synaptic activity (James et al., 2015). Our results also showed that the seizure severity is reduced in the VPA- subgroup. This effect could be interpreted as an apparent protection of a subgroup of VPA exposed rats against PTZ. Sobrian & Nandedkar (1986) analyzed whether sub-chronic prenatal exposure to VPA could protect or sensitize against PTZ-induced convulsions in 34–38 days old rats. These authors did not use the VPA as an experimental model of autism. Actually, they used a non-teratogenic dose of VPA (20 mg/kg) and the drug was injected from 15th to 20th embryonic day. Their results are similar to our findings in P14 rat pups, since VPA exposed rats exhibited a sexually dimorphic response to PTZ-induced convulsions (50 mg/kg): VPA prenatal exposure protected females from generalized clonic-tonic convulsions, whereas duration of this seizure was longer in VPA male offspring than in controls (Sobrian & Nandedkar, 1986). These authors speculate that the protective effect conferred by prenatal valproate may be due to an increase in fetal levels of GABA, changes in drug metabolism promoting an increased degradation of PTZ, or an altered PTZ binding capacity (Sobrian & Nandedkar, 1986). All these changes may also explain the protective effect observed in our study. However, there is not yet a clear explanation for the decreased PTZ-seizure susceptibility observed after prenatal exposure to VPA.

As previously mentioned, the VPA+ subgroup had the same seizure incidence and severity than the control group, but displayed higher duration of the GTCS and the tonic phase of that seizure, and an increased number of rats had SE; the VPA− subgroup did not displayed GTCS. A possible explanation for this dual effect of VPA treatment is the intrauterine fetal position. Fetuses from rats treated with cocaine had different brain drug levels depending on their localization; fetuses near to cervix had higher levels when compared with fetuses distal from it (Del Campo & Ginther, 1972). This effect could be associated with the uterine blood flow, since blood flows predominantly from the cervix to the ovaries in pregnant rats (Lipton et al., 1998). In the same context, a recent paper showed that prenatal exposure to letrozole produces two subpopulations of rats with different sex preference (Olvera-Hernández, Chvira & Fernández-Guasti, 2015). To explain this observation, the authors suggested that the fetuses were exposed in utero to a different amount of drug. This could also explain the various VPA teratogenic effects observed in the same litter in this present study. Interestingly, heterogeneity has also been demonstrated in humans with ASD (Oberman et al., 2010). In this study, intracortical inhibition was measured in people with ASD and a subgroup with reduced inhibition was reported, which could be related with the broad spectrum of behaviors observed in this condition (Oberman et al., 2010).

The VPA+ subgroup was mainly made of male rats and increased seizures susceptibility was observed in male but not in female rat pups. Sex differences can play a key factor in the development of psychological disorders such as autism (Werling & Geschwind, 2013). In this context, autism is more common in men than women (Fombonne, 2009; Mandy et al., 2012). It has been proposed that a dysregulation of estrogen receptor signaling may contribute to sex differences in autism (Crider et al., 2014). Interestingly, our work group recently showed that prenatal VPA induces gender-dependent changes in androgen receptor expression in the developing rat cerebellum (Perez-Pouchoulen et al., 2016), which may contribute to explain the differences in seizure susceptibility observed in this study. In the experiments reported by Kim et al. (2013), a lower seizure threshold was found after VPA exposure; only male rats were used. Thus, our results suggest that in the VPA-exposed rat model of ASD, males could be more sensitive to a possible reduction in the brain inhibitory function and, consequently, VPA males could be more susceptible to develop seizures. It is known that glutamic acid decarboxylase (GAD67) expression in VPA-exposed female hippocampus is higher compared with male offspring (Kim et al., 2013). However, it has recently been shown that adult VPA rats had increased mRNA expression for GABA transporter 1 in the amygdala and decreased levels of GAD67 and GAD65 in the cerebellum compared to control animals, in both genders (Olexová, Štefánik & Kršková, 2016). These data suggest that the mechanisms responsible for gender specificity in ASD and epilepsy can involve different brain areas. Additionally, while using the same experimental model of autism, others found a gender difference in postnatal behavior, endocrine activity, and immune system function in VPA rats; further, some of these parameters were similar in VPA and control female rats (Schneider et al., 2008). Several studies have reported that the prevalence of autism is higher in boys than in girls; however autistic girls have a greater incidence of epilepsy (Bolton et al., 2011; Danielsson et al., 2005; Hughes & Melyn, 2005; Kanner, 1943).

Klioueva et al. (2001) found differences in PTZ-induced seizure severity in rats at different ages, pointing that the immaturity of the brain could be a determining factor. In that study, 10-days-old rat pups reached seizures with a shorter latency than older rats, their pattern of seizures was relatively immature, and fully generalized seizures in this age group failed to occur (Klioueva et al., 2001). In immature brains, the GABAergic system is not completely developed and its inefficiency could result in increased susceptibility to seizures. In fact, the absence of inhibitory control on excitatory processes at an early age increases the vulnerability of the developing neocortex to seizure activity during postnatal ontogenesis (Ben-Ari et al., 1989; Klioueva et al., 2001). Thus, VPA+ rats may be more susceptible to PTZ effects, due to an under-development of the GABAergic system. Our data support that prenatal exposure of VPA favors seizure activity, perhaps due to an imbalance between the inhibitory and excitatory systems, probably involving GABA. Additionally, a recent study demonstrated that prenatal VPA exposure in rats induces changes in astrocytic parameters in 15-days-old male rats, including an increase of glutamine synthetase (GS) activity and a decrease in the expression of the glutamate transporters GLT1 (Bristot Silvestrin et al., 2013). An increase in GS activity could result in metabolic changes affecting the production of glutamate, whereas the reduction of GLT1 could result in increased extracellular concentration of glutamate, which in turn could augment neuronal excitability. These effects could be associated with the higher seizure susceptibility observed in the VPA+ subgroup and also in autism patients (Bolton et al., 2011; Danielsson et al., 2005; Gabis, Pomeroy & Andriola, 2005; Hara, 2007; Hughes & Melyn, 2005; Tebartz van Elst et al., 2014). Our results replicate data reported in several epidemiological studies showing comorbidity between autism and epilepsy (Bolton et al., 2011; Danielsson et al., 2005; Gabis, Pomeroy & Andriola, 2005; Hara, 2007; Hughes & Melyn, 2005) and specifically, the manifestation of generalized tonic clonic seizures (Bolton et al., 2011; Hara, 2007; Hughes & Melyn, 2005). However, our data do not infer that prenatal exposure to VPA and seizure susceptibility in the offspring is triggered by an alteration in the GABAergic system, since we have not data about neurotransmission and long term EEG-video monitoring was not performed. However, these effects could be caused by a dysfunction of the GABAergic system.

On the other hand, in utero VPA exposure may affect normal rat brain maturation, affecting importantly the GABAergic system and modifying seizure susceptibility as seen in this study. The excitatory or inhibitory effect of GABA during neurodevelopment depends on the electrochemical gradient of Cl− across the neuronal plasma membrane, which is regulated by cation-chloride cotransporters: KCC2 drive net Cl− extrusion whereas NKCC1 uptakes Cl− by using the K+ and Na+ gradients which, in turn, are generated by the Na-K ATPase (reviewed in Löscher, Puskarjov & Kaila, 2013). Intracellular Cl− concentration is high in immature neurons, which is attributable to NKCC1 and responsible for depolarizing effects of GABA. Later, there is a developmental up-regulation of KCC2, which renders the equilibrium potential of Cl− hyperpolarizing and promotes the inhibitory effect of GABA (reviewed in Löscher, Puskarjov & Kaila, 2013). It has been proposed that an abnormal functional expression of KCC2 and NKCC1 might promote GABAergic excitation, which is relevant for epilepsy (Huberfeld et al., 2007; Löscher, Puskarjov & Kaila, 2013) as well as autism (Hadjikhani et al., 2015; Lemonnier et al., 2012). Prenatal VPA may modify the normal expression and functioning of NKCC1 and KCC2, which may alter the inhibitory effect of GABA at P14, when rats were tested for PTZ convulsions and produce the different degree of susceptibility to PTZ− induced convulsions observed in this study.

We found that more rats in the VPA+ subgroup developed multiple GTCS compared to the control group, and the duration of GTCS and the tonic phase were longer in VPA+ rats compared to controls. It is known that brainstem plays an important role in the expression of the tonic seizures induced by PTZ (Eells et al., 2004; Franco-Pérez, Ballesteros-Zebadúa & Manjarrez-Marmolejo, 2015) and recently Dubiel & Kulesza (2016) showed significantly more c-Fos-positive neurons in the auditory brainstem of VPA-exposed rats. These changes were associated with a hyper-responsiveness to sounds and a disrupted mapping of sound frequencies at postnatal day 28. It is possible that prenatal VPA has increased neuronal activity in specific brainstem areas facilitating the induction of GTCS in the VPA+ subgroup.

Our study showed no differences between controls and VPA exposed rats in the Li-Pilo model with regard to seizure severity, latency to SE and duration of SE. It is known that the cholinergic neuronal network is functional by the end of the second week of life in rats (Coyle & Yamamura, 1976). However, it was recently found that prenatal exposure of VPA (300 mg/kg) induced an increase of acetylcholinesterase expression and activity in the frontal cortex of mice at postnatal day 35 (Kim et al., 2014). This dysregulation of cholinergic neuronal development may also be present in VPA rats and may explain the absence of differences between VPA and control groups in pilocarpine-induced SE, considering that the brain of VPA rats have adapted to the increased cholinergic activity.

## Conclusion

The present study shows that prenatal VPA modifies PTZ seizure susceptibility in infant rats, identifying subpopulations with higher susceptibility (VPA+) and other with lower susceptibility (VPA−). These findings may contribute to a better understanding of the comorbidity between autism and epilepsy.

## Supplemental Information

10.7717/peerj.2709/supp-1 Supplemental Information 1 Raw data for seizure behavioral analysis.

## Additional Information and Declarations

### Data Deposition

The following information was supplied regarding data availability: The raw data has been supplied as Supplemental Dataset Files.

## References

[ref-1] Arndt TL, Stodgell CJ, Rodier PM (2005). The teratology of autism. International Journal of Developmental Neuroscience.

[ref-2] Ben-Ari Y, Cherubini E, Corradetti R, Gaiarsa JL (1989). Giant synaptic potentials in immature rat CA3 hippocampal neurones. Journal of Physiology.

[ref-3] Bolton PF, Carcani-Rathwell I, Hutton J, Goode S, Howlin P, Rutter M (2011). Epilepsy in autism: features and correlates. The British Journal of Psychiatry.

[ref-4] Bristot Silvestrin R, Bambini-Junior V, Galland F, Daniele Bobermim L, Quincozes-Santos A, Torres Abib R, Zanotto C, Batassini C, Brolese G, Gonçalves CA, Riesgo R, Gottfried C (2013). Animal model of autism induced by prenatal exposure to valproate: altered glutamate metabolism in the hippocampus. Brain Research.

[ref-5] Bromley RL, Mawer G, Clayton-Smith J, Baker GA, Liverpool Manchester Neurodevelopment Group (2008). Autism spectrum disorders following in utero exposure to antiepileptic drugs. Neurology.

[ref-6] Bromley RL, Mawer GE, Briggs M, Cheyne C, Clayton-Smith J, García-Fiñana M, Kneen R, Lucas SB, Shallcross R, Baker GA, Liverpool Manchester Neurodevelopment Group (2013). The prevalence of neurodevelopmental disorders in children prenatally exposed to antiepileptic drugs. Journal of Neurology, Neurosurgery, and Psychiatry.

[ref-7] Christianson AL, Chesler N, Kromberg JGR (1994). Fetal valproate syndrome: clinical and neuro-developmental features in two sibling pairs. Developmental Medicine & Child Neurology.

[ref-8] Coyle JT, Yamamura RI (1976). Neurochemical aspects of the ontogenesis of cholinergic neurons in the rat brain. Brain Research.

[ref-9] Crider A, Thakkar R, Ahmed AO, Pillai A (2014). Dysregulation of estrogen receptor beta (ERβ), aromatase (CYP19A1), and ER co-activators in the middle frontal gyrus of autism spectrum disorder subjects. Molecular Autism.

[ref-10] Danielsson S, Gillberg IC, Billstedt E, Gillberg C, Olsson I (2005). Epilepsy in young adults with autism: a prospective population-based follow-up study of 120 individuals diagnosed in childhood. Epilepsia.

[ref-11] Del Campo CH, Ginther OJ (1972). Vascular anatomy of the uterus and ovaries and the unilateral luteolytic effect of the uterus: guinea pigs, rats, hamsters and rabbits. American Journal of Veterinary Research.

[ref-12] DeLorenzo RJ, Hauser WA, Towne AR, Boggs JG, Pellock JM, Penberthy L, Garnett L, Fortner CA, Ko D (1996). A prospective, population-based epidemiologic study of status epilepticus in Richmond, Virginia. Neurology.

[ref-13] Dubiel A, Kulesza RJ (2016). Prenatal valproic acid exposure disrupts tonotopic c-Fos expression in the rat brainstem. Neuroscience.

[ref-14] Eells JB, Clough RW, Browning RA, Jobe PC (2004). Comparative fos immunoreactivity in the brain after forebrain, brainstem, or combined seizures induced by electroshock, pentylenetetrazol, focally induced and audiogenic seizures in rats. Neuroscience.

[ref-15] Favre MR, Barkat TR, LaMendola D, Khazen G, Markram H, Markram K (2013). General developmental health in the VPA-rat model of autism. Frontiers in Behavioral Neuroscience.

[ref-16] Fisher RS, Acevedo C, Arzimanoglou A, Bogacz A, Cross JH, Elger CE, Engel J, Forsgren L, French JA, Glynn M, Hesdorffer DC, Lee BI, Mathern GW, Moshé SL, Perucca E, Scheffer IE, Tomson T, Watanabe M, Wiebe S (2014). ILAE official report: a practical clinical definition of epilepsy. Epilepsia.

[ref-17] Foley AG, Gannon S, Rombach-Mullan N, Prendergast A, Barry C, Cassidy AW, Regan CM (2012). Class I histone deacetylase inhibition ameliorates social cognition and cell adhesion molecule plasticity deficits in a rodent model of autism spectrum disorder. Neuropharmacology.

[ref-18] Fombonne E (2009). Epidemiology of pervasive developmental disorders. Pediatric Research.

[ref-19] Franco-Pérez J, Ballesteros-Zebadúa P, Manjarrez-Marmolejo J (2015). Unilateral microinjection of carbenoxolone into the pontis caudalis nucleus inhibits the pentylenetetrazole-induced epileptiform activity in rats. Neuroscience Letters.

[ref-20] Fried S, Kozer E, Nulman I, Einarson TR, Koren G (2004). Malformation rates in children of women with untreated epilepsy. Drug Safety.

[ref-21] Gabis L, Pomeroy J, Andriola MR (2005). Autism and epilepsy: cause, consequence, comorbidity, or coincidence?. Epilepsy & Behavior.

[ref-22] Govoni V, Fallica E, Monetti VC, Guerzoni F, Faggioli R, Casetta I, Granieri E (2008). Incidence of status epilepticus in southern Europe: a population study in the health district of Ferrara, Italy. European Neurology.

[ref-23] Haas KZ, Sperber EF, Moshe SL (1990). Kindling in developing animals: expression of severe seizures and enhanced development of bilateral foci. Brain research. Developmental Brain Research.

[ref-24] Hadjikhani N, Zürcher NR, Rogier O, Ruest T, Hippolyte L, Ben-Ari Y, Lemonnier E (2015). Improving emotional face perception in autism with diuretic bumetanide: a proof-of-concept behavioral and functional brain imaging pilot study. Autism.

[ref-25] Hara H (2007). Autism and epilepsy: a retrospective follow-up study. Brain & Development.

[ref-26] Haut SR, Velísková J, Moshé SL (2004). Susceptibility of immature and adult brains to seizure effects. The Lancet Neurology.

[ref-27] Huberfeld G, Wittner L, Clemenceau S, Baulac M, Kaila K, Miles R, Rivera C (2007). Perturbed chloride homeostasis and GABAergic signaling in human temporal lobe epilepsy. Journal of Neuroscience.

[ref-28] Hughes JR, Melyn M (2005). EEG and seizures in autistic children and adolescents: further findings with therapeutic implications. Clinical EEG and Neuroscience.

[ref-29] James EJ, Gu J, Ramirez-Vizcarrondo CM, Hasan M, Truszkowski TL, Tan Y, Oupravanh PM, Khakhalin AS, Aizenman CD (2015). Valproate-induced neurodevelopmental deficits in *Xenopus laevis* tadpoles. Journal of Neuroscience.

[ref-30] Kanner L (1943). Autistic disturbances of affective contact. Nervous Child.

[ref-31] Kim JW, Seung H, Kwon KJ, Ko MJ, Lee EJ, Oh HA, Choi CS, Kim KC, Gonzales EL, You JS, Choi D-H, Lee J, Han S-H, Yang SM, Cheong JH, Shin CY, Bahn GH (2014). Subchronic treatment of donepezil rescues impaired social, hyperactive, and stereotypic behavior in valproic acid-induced animal model of autism. PLoS ONE.

[ref-32] Kim KC, Kim P, Go HS, Choi CS, Park JH, Kim HJ, Jeon SJ, Dela Pena IC, Han SH, Cheong JH, Ryu JH, Shin CY (2013). Male-specific alteration in excitatory post-synaptic development and social interaction in prenatal valproic acid exposure model of autism spectrum disorder. Journal of Neurochemistry.

[ref-33] Kim KC, Kim P, Go HS, Choi CS, Yang SI, Cheong JH, Shin CY, Ko KH (2011). The critical period of valproate exposure to induce autistic symptoms in Sprague-Dawley rats. Toxicology Letters.

[ref-34] Klioueva IA, van Luijtelaar ELJM, Chepurnova NE, Chepurnov SA (2001). PTZ-induced seizures in rats: effects of age and strain. Physiology & Behavior.

[ref-35] Lemonnier E, Degrez C, Phelep M, Tyzio R, Josse F, Grandgeorge M, Hadjikhani N, Ben-Ari Y (2012). A randomised controlled trial of bumetanide in the treatment of autism in children. Translational Psychiatry.

[ref-36] Lipton JW, Robie HC, Ling Z, Weese-Mayer DE, Carvey PM (1998). The magnitude of brain dopamine depletion from prenatal cocaine exposure is a function of uterine position. Neurotoxicology and Teratology.

[ref-37] Löscher W, Puskarjov M, Kaila K (2013). Cation-chloride cotransporters NKCC1 and KCC2 as potential targets for novel antiepileptic and antiepileptogenic treatments. Neuropharmacology.

[ref-38] Mandy W, Chilvers R, Chowdhury U, Salter G, Seigal A, Skuse D (2012). Sex differences in autism spectrum disorder: evidence from a large sample of children and adolescents. Journal of Autism and Developmental Disorders.

[ref-39] Meador K, Reynolds MW, Crean S, Fahrbach K, Probst C (2008). Pregnancy outcomes in women with epilepsy: a systematic review and meta-analysis of published pregnancy registries and cohorts. Epilepsy Research.

[ref-40] Moore SJ, Turnpenny P, Quinn A, Glover S, Lloyd DJ, Montgomery T, Dean JCS (2000). A clinical study of 57 children with fetal anticonvulsant syndromes. Journal of Medical Genetics.

[ref-62] National Research Council of the National Academies (2011). Guide for the Care and Use of Laboratory Animals.

[ref-41] Oberman L, Ifert-Miller F, Najib U, Bashir S, Woollacott I, Gonzalez-Heydrich J, Picker J, Rotenberg A, Pascual-Leone A (2010). Transcranial magnetic stimulation provides means to assess cortical plasticity and excitability in humans with fragile X syndrome and autism spectrum disorder. Frontiers in Synaptic Neuroscience.

[ref-42] Olexová L, Štefánik P, Kršková L (2016). Increased anxiety-like behaviour and altered GABAergic system in the amygdala and cerebellum of VPA rats––an animal model of autism. Neuroscience Letters.

[ref-43] Olvera-Hernández S, Chvira R, Fernández-Guasti A (2015). Prenatal letrazole produces a subpopulation of male rats with same-sex preference and arousal as well as female sexual behavior. Physiology & Behavior.

[ref-44] Perez-Pouchoulen M, Miquel M, Saft P, Brug B, Toledo R, Hernandez ME, Manzo J (2016). Prenatal exposure to sodium valproate alters androgen receptor expression in the developing cerebellum in a region and age specific manner in male and female rats. International Journal of Developmental Neuroscience.

[ref-45] Phiel CJ, Zhang F, Huang EY, Guenther MG, Lazar MA, Klein PS (2001). Histone deacetylase is a direct target of valproic acid, a potent anticonvulsant, mood stabilizer, and teratogen. Journal of Biological Chemistry.

[ref-46] Pohl M, Mares P (1987). Flunarizine influences metrazol-induced seizures in developing rats. Epilepsy Research.

[ref-47] Puig-Lagunes AA, Velazco-Cercas E, Zamora-Bello II, Beltrán-Parrazal L, Morgado-Valle C, Manzo J, López-Meraz ML (2015). Malformaciones congénitas en ratas expuestas prenatalmente al ácido valproico y su relación con el número de células de Purkinje. Revista Mexicana de Neurociencia.

[ref-48] Rapin I, Tuchman RF (2008). Autism: definition, neurobiology, screening, diagnosis. Pediatric Clinics of North America.

[ref-49] Rodier PM, Ingram JL, Tisdale B, Nelson S, Romano J (1996). Embryological origin for autism: developmental anomalies of the cranial nerve motor nuclei. Journal of Comparative Neurology.

[ref-50] Saft P, Toledo-Cardenas R, Coria-Avila GA, Perez-Pouchoulen M, Brug B, Hernandez ME, Manzo J (2014). Characterization of four types of tail abnormalities in rats treated prenatally with valproic acid. Revista Eneurobiología.

[ref-51] Schneider T, Przewlocki R (2005). Behavioral alterations in rats prenatally exposed to valproic acid: animal model of autism. Neuropsychopharmacology.

[ref-52] Schneider T, Roman A, Basta-Kaim A, Kubera M, Budziszewska B, Schneider K, Przewłocki R (2008). Gender-specific behavioral and immunological alterations in an animal model of autism induced by prenatal exposure to valproic acid. Psychoneuroendocrinology.

[ref-53] Sobrian SK, Nandedkar AKN (1986). Prenatal antiepileptic drug exposure alters seizure susceptibility in rats. Pharmacology Biochemistry and Behavior.

[ref-54] Suchomelova L, Baldwin RA, Kubova H, Thompson KW, Sankar R, Wasterlain CG (2006). Treatment of experimental status epilepticus in immature rats: dissociation between anticonvulsant and antiepileptogenic effects. Pediatric Research.

[ref-55] Suchomelova L, Lopez-Meraz ML, Niquet J, Kubova H, Wasterlain CG (2015). Hyperthermia aggravates status epilepticus-induced epileptogenesis and neuronal loss in immature rats. Journal of Neuroscience.

[ref-56] Tebartz van Elst L, Maier S, Fangmeier T, Endres D, Mueller GT, Nickel K, Ebert D, Lange T, Hennig J, Biscaldi M, Riedel A, Perlov E (2014). Disturbed cingulate glutamate metabolism in adults with high-functioning autism spectrum disorder: evidence in support of the excitatory/inhibitory imbalance hypothesis. Molecular Psychiatry.

[ref-57] Turski L, Ikonomidou C, Turski WA, Bortolotto ZA, Cavalheiro EA (1989). Cholinergic mechanisms and epileptogenesis: the seizures induced by pilocarpine: a novel experimental model of intractable epilepsy. Synapse.

[ref-58] Velisek L, Kubova H, Polh M, Stankova L, Mareš P, Schickerova R (1992). Pentylenetetrazol-induced seizures in rats: an ontogenetic study. Naunyn-Schmiedeberg’s Archives of Pharmacology.

[ref-59] Wasterlain CG, Chen J, Wasterlain CG, Treiman DM (2006). Definition and classification of status epilepticus. Status Epilepticus: Mechanisms and Management.

[ref-60] Werling DM, Geschwind DH (2013). Sex differences in autism spectrum disorders. Current Opinion in Neurology.

[ref-61] Williams PG, Hesh JH (1997). A male with fetal valproate syndrome and autism. Developmental Medicine & Child Neurology.

